# Innovative dressing and securement of tunneled central venous access devices in pediatrics: a pilot randomized controlled trial

**DOI:** 10.1186/s12885-017-3606-9

**Published:** 2017-08-30

**Authors:** Amanda J. Ullman, Tricia Kleidon, Victoria Gibson, Craig A. McBride, Gabor Mihala, Marie Cooke, Claire M. Rickard

**Affiliations:** 1 School of Nursing and Midwifery, Griffith University, Nathan, Queensland Australia; 2 Alliance for Vascular Access Teaching and Research Group, Menzies Health Institute Queensland, Nathan, Queensland Australia; 3 Children’s Health Queensland, Lady Cilento Children’s Hospital, South Brisbane, Queensland Australia; 4 School of Medicine, University of Queensland, Herston, Queensland Australia; 5 School of Medicine, Griffith University, Gold Coast, Queensland Australia; 6 Centre for Applied Health Economics, Menzies Health Institute Queensland , Nathan, Queensland Australia

**Keywords:** Central venous catheter, Dressing, Randomized controlled trial, Evidence-based care, Pediatrics

## Abstract

**Background:**

Central venous access device (CVAD) associated complications are a preventable source of patient harm, frequently resulting in morbidity and delays to vital treatment. Dressing and securement products are used to prevent infectious and mechanical complications, however current complication rates suggest customary practices are inadequate. The aim of this study was to evaluate the feasibility of launching a full-scale randomized controlled efficacy trial of innovative dressing and securement products for pediatric tunneled CVAD to prevent complication and failure.

**Methods:**

An external, pilot, four-group randomized controlled trial of standard care (bordered polyurethane dressing and suture), in comparison to integrated securement-dressing, suture-less securement device, and tissue adhesive was undertaken across two large, tertiary referral pediatric hospitals in Australia. Forty-eight pediatric participants with newly inserted tunneled CVADs were consecutively recruited. The primary outcome of study feasibility was established by elements of eligibility, recruitment, attrition, protocol adherence, missing data, parent and healthcare staff satisfaction and acceptability, and effect size estimates for CVAD failure (cessation of function prior to completion of treatment) and complication (associated bloodstream infection, thrombosis, breakage, dislodgement or occlusion). Dressing integrity, product costs and site complications were also examined.

**Results:**

Protocol feasibility was established. CVAD failure was: 17% (2/12) integrated securement-dressing; 8% (1/13) suture-less securement device; 0% tissue adhesive (0/12); and, 0% standard care (0/11). CVAD complications were: 15% (2/13) suture-less securement device (CVAD associated bloodstream infection, and occlusion and partial dislodgement); 8% (1/12) integrated securement-dressing (partial dislodgement); 0% tissue adhesive (0/12); and, 0% standard care (0/11). One CVAD-associated bloodstream infection occurred, within the suture-less securement device group. Overall satisfaction was highest in the integrated securement-dressing (mean 8.5/10; standard deviation 1.2). Improved dressing integrity was evident in the intervention arms, with the integrated securement-dressing associated with prolonged time to first dressing change (mean days 3.5).

**Conclusions:**

Improving the security and dressing integrity of tunneled CVADs is likely to improve outcomes for pediatric patients. Further research is necessary to identify novel, effective CVAD securement to reduce complications, and provide reliable vascular access for children.

**Trial registration:**

ACTRN12614000280606; prospectively registered on 17/03/2014.

## Background

Children undergoing treatment for oncological and hematological conditions are some of the most vulnerable patients in hospital settings. Functioning, complication-free vascular access for anti-cancer therapies, antibiotics, nutrition and blood sampling is vital to their treatment and survival. Tunneled, cuffed central venous access devices (CVADs), commonly referred to by their trade names, Hickman® or Broviac® catheters, are inserted for children requiring multiple or frequent infusions of irritant or vesicant fluids over prolonged periods of time (≥3 months) [1]. The pediatric population requiring this type of central device is primarily receiving treatment for oncological (e.g. neuroblastoma), hematological (e.g. aplastic anemia) or gastroenterological (e.g. intestinal failure) conditions [2, 3].

Tunneled, cuffed CVADs incorporate a short Dacron cuff designed to inhibit ascending migration of organisms from the skin, and to stimulate tissue growth around the cuff to anchor the device [4]. However Dacron cuff adhesion takes up to four weeks [4, 5], longer when the patient is compromised, placing the CVAD at significant risk of dislodgement or infection prior to that time. While historically considered a ‘safe’ device, a recent meta-analysis [6] demonstrated 29% of pediatric tunneled cuffed CVADs failed prior to completion of therapy (0.86 per 1,000 catheter days), with 20% developing a CVAD-associated bloodstream infection (BSI) (1.13 per 1,000 catheter days), and 7% dislodging completely (0.24 per 1,000 catheter days).

A key strategy to decrease the risk of CVAD-associated complication is to ensure the insertion wound is adequately covered to prevent infection, and the device secured to prevent internal and external motion [7]. Traditionally CVAD insertion sites were covered with sterile gauze and tape, with polyurethane dressings becoming prominent practice in the 1990s [8]. The devices are also internally and/or externally sutured, with these sutures removed, or dissolving, at around 7–10 days. The current failure and complication rates associated with CVADs suggest that habitual practices of their dressing and securement are inadequate.

A recent Cochrane systematic review [9] highlighted the dearth of literature to support clinical decision making in the area of CVAD securement, considering the range of available products. Previous randomized controlled trials (RCTs) of securement and dressing products for tunneled, cuffed CVAD are dated [10, 11] and have limited their study population to adults [10, 12–14]. No previous RCTs have focused on children or examined the issue of CVAD security. The physiology and pathophysiology of children requiring these devices necessitates a specialist focus [15].

Newer CVAD securement and dressing products are available, which may be superior to traditional methods. Integrated securement-dressings (ISDs) incorporate a strengthened securement system across the entire polyurethane dressing, over and underneath the CVAD body [7]. ISDs also surround the polyurethane with an absorbent layer, to maintain dressing integrity when exposed to insertion site exudate. Suture-less securement devices (SSD) comprise of soft footplates with fastening clasps of hard plastic or soft Velcro to reduce movement and catheter rotation [16]. Tissue adhesive (TA), a medical grade ‘superglue’ (cyanoacrylate), can provide strength and hemostatic properties [17], which may be beneficial for patients experiencing large amounts of post-insertion exudate due to underlying pathologies. However, it is not known whether these new products are more effective at reducing pediatric CVAD failure and complication, in comparison to traditional care. In order to reduce CVAD associated complications in the pediatric population, RCTs of CVAD securement products are necessary to provide true estimates of relative effectiveness and inform practice [18]. Prior to undertaking large efficacy trials, external pilot studies are necessary to examine issues of research feasibility including intervention acceptability, compliance and recruitment [18].

## Methods

### Aims

The primary aim of this research was to evaluate the feasibility of launching a full-scale randomized controlled efficacy trial of pediatric tunneled CVAD securement and dressing, using pre-defined feasibility criteria for recruitment, retention, protocol fidelity and product acceptability. The secondary aim was to compare the effectiveness of dressings and securement products on tunneled CVAD complications and failure due to infection, occlusion, dislodgement, thrombosis, or breakage, for children in acute care facilities.

### Design

This study was a four-arm, external pilot randomized controlled trial. Prior to study commencement the trial was registered with the Australian Clinical Trial Registry (ACTRN12614000280606), including a published protocol [19].

### Study setting

The trial was commenced at the Royal Children’s Hospital, Brisbane, Australia; and, after local hospital mergers, completed at the larger Lady Cilento Children’s Hospital, Brisbane, Australia. These are tertiary level, specialist pediatric teaching hospitals, providing health services to children and young people from birth to 18 years of age throughout Queensland, northern New South Wales and the Pacific Rim.

### Sample

Participants who met the inclusion criteria were consecutively recruited: requiring a tunneled, cuffed CVAD; less than 18 years of age; would remain hospital inpatients for greater than 24 h; and informed consent to participate. Patients were not eligible if they had a current bloodstream infection, consent givers were non-English speakers without an interpreter, the CVADs were to be inserted through diseased, burned, scarred or extremely diaphoretic skin, had a known allergy to the study products, had a current skin tear, or had previously been enrolled in the study within the current hospital admission.

Twelve participants were recruited per intervention group, with a final sample of 48 participants. These sample size calculations were developed in accordance with the recommendations by SA Julious [20] and M Hertzog [21]; to facilitate accurate estimates of effect size while minimizing unnecessary costs, time and recruitment of future definitive study participants, where little data are available to base a sample size calculation.

### Interventions

Participants were randomized to receive CVAD dressing and securement by:

- Standard care: Suture (Prolene®; Ethicon, New Jersey); and bordered polyurethane (BPU) dressing (Tegaderm® 1655 or 1616 dependent upon participant size; 3M, St Paul);
- Suture-less securement device (SSD): Suture (Prolene®; Ethicon, New Jersey) (suture was deemed necessary due to large tunnel wound); Suture-less securement device (staff preference of StatLock® VFDSSP; Bard, Georgia or GripLok® 3601CVC; TIDI, Neenah WI); and BPU dressing (Tegaderm® 1655 or 1616; 3M, St Paul);
- Tissue adhesive (TA): One-two drops of Tissue adhesive (Histoacryl®; B. Braun, Germany) at exit wound and under catheter bifurcation; and BPU dressing (Tegaderm® 1655 or 1616; 3M, St Paul);
- Integrated securement-dressings (ISD): Suture (Prolene®; Ethicon, New Jersey); and ISD (SorbaView SHIELD® SV254; Centurion Medical Products, Williamston).

**Fig. 1:**
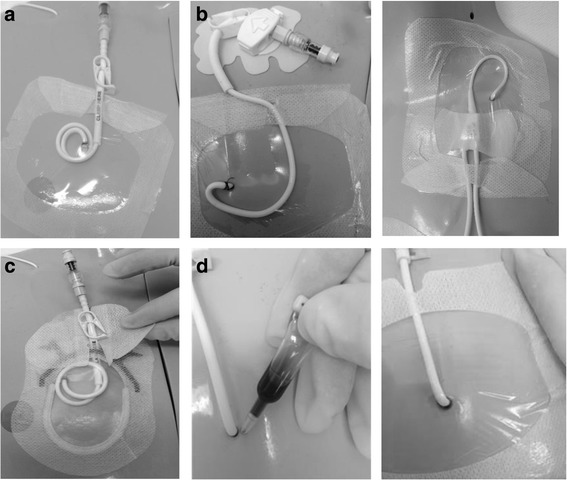
Intervention products. **a** Bordered polyurethane and suture; **b** Bordered polyurethane, suture and suture-less securement device; **c** Integrated securement dressing and suture; **d** Bordered polyurethane and tissue adhesive (no suture)

The application of these intervention arms can be seen in Fig. 1. The intervention arms were developed taking into consideration current local practice, best available evidence and the safety of all participants.

### Outcomes

The primary outcome was feasibility of a full efficacy trial, established by composite analysis of elements of eligibility, recruitment, attrition, protocol adherence, missing data, parent and healthcare staff satisfaction, and effect size estimates to allow sample size calculations [21–23]. Sample size estimates were to be based upon the proportion of CVAD failure (cessation of catheter function prior to completion of therapy [6]), and CVAD complication (a composite of CVAD-associated BSI (CABSI), local infection, occlusion, dislodgement, venous thrombosis and breakage). Each CVAD complication was defined in accordance with best practice guidance [3, 6, 24, 25].

Secondary outcomes included the individual CVAD complications, CVAD-related BSI [24], securement-dressing failure, time to first dressing change, skin complications and direct product costs [26]. Full definitions of the primary and secondary outcomes can be found within the published protocol [19]. Diagnoses of CABSI and CVAD-related BSI outcomes were by an independent, blinded infectious diseases specialist. Similarly, ultrasound for the identification of symptomatic venous thrombosis was requested by the clinical team coordinating the participants’ care, with diagnosis made by an independent, blinded radiologist using standard departmental protocols.

### Study Procedures

The research nurse (ReN) screened patients daily via theatre bookings, then obtained written informed consent, and performed randomization. Randomization was evenly distributed 1:1:1:1 between study groups with randomly varying block sizes, using a web-based service (https://www151.griffith.edu.au/random) that ensured concealment. The ReN checked patients daily to inspect the CVAD and dressing securement products, collect data, and to ensure safety of the study participants. Participants were included in the trial until four weeks after CVAD insertion, or to study withdrawal, removal of the CVAD, or hospital discharge, if these occurred before four weeks. CVAD securement and dressings were not amenable to blinding of patients, clinical staff or ReNs.

De-identified data collection was undertaken via REDCap (Research Electronic Data CAPture http://project-redcap.org/). The ReN collected data on primary and secondary outcomes using the pre-defined criteria. Demographic data were collected to assess success of randomization, describe the participant group and enable comparisons to inform future generalizability. Data were also collected regarding clinical characteristics known to increase the risk of CVAD complication and dressing integrity, including age, comorbidities, immune-compromise, CVAD utility, skin condition, insertion site and technique [2, 3, 27–31]. At CVAD insertion and removal (or within 24 h), the ReN asked the parents or caregivers, and healthcare staff for their perceived satisfaction with the intervention products (numeric rating scale 0–10 with increased positivity with higher scores).

#### CVAD procedures

All non-antimicrobial tunneled, cuffed CVADs (Cook®; Cook Medical; Bloomington) were inserted in an operating theatre by a qualified consultant pediatric surgeon, or a surgeon in an approved pediatric surgical program, and managed by clinical staff in accordance with state and hospital policy [32–34]. The inserter chose the CVAD characteristics based on clinical judgement of patient needs, and then applied the allocated products [35]. Local hospital policy directed site decontamination at insertion with aqueous Betadine (10% povidone-iodine) and no routine antibiotic prophylaxis at insertion [34]. To maximize generalizability, clinical nursing staff (not ReNs) changed study products weekly and as clinically indicated (e.g. interruption of dressing integrity), with education assistance regarding dressing application provided by the ReN. Product replacements/reinforcements, including tape were recorded. An absorbent dressing, such as sterile gauze, was used alternatively independent from the treatment arm temporarily if the CVAD site was bleeding or oozing [5, 32], with such use and its duration, recorded.

All other CVAD management procedures were as per hospital policy including the use of 2% chlorhexidine gluconate in 70% alcohol for insertion site decontamination during dressing change, frequency and volume of flushing, negative or neutral displacement mechanical valve needleless connectors, intravenous medication administration and administration set changes [33]. Clinical staff undertook blood and CVAD tip cultures on suspicion of infection, as per standard hospital and pathology protocols [32, 36, 37] .

### Statistical Analyses

Descriptive statistics (counts, percentages) were used to ascertain the primary outcome of feasibility for the larger trial. All randomized patients were analyzed on an Intention to Treat (ITT) basis [38]. Comparability of groups at baseline was described across demographic, clinical and device characteristics. Incidence rates (IR) of CVAD device failure and complication (per 1,000 catheter days) were used to summarize the impact of the intervention; with differences evaluated by calculating 95% confidence intervals.

Kaplan-Meier survival curves (with log rank test) were used to compare CVAD failure, complication, and first dressing duration over time. Standard data cleaning of outlying figures, missing, and implausible data was undertaken prior to analysis. Missing values were not imputed. P values of <0.1 were evaluated as indicating some evidence against a null hypothesis, and values <0.05 were considered statistically significant. Stata [39] was used for all analyses.

### Ethics

Ethics approval for the trial was gained from the Children’s Health Services Queensland (HREC/13/QRCH/181) and Griffith University (NRS/10/14/HREC) Human Research Ethics Committees (HREC). Written informed consent from parents or legal guardians was gained prior to enrolment. Child assent was also gained, where appropriate.

## Results

**Fig. 2:**
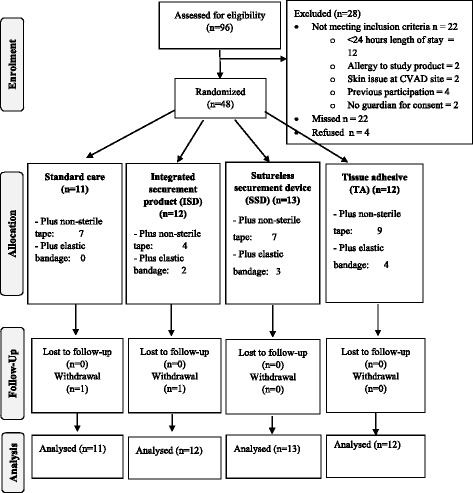
CONSORT flow chart of study participants

The study recruited for 21 months, commencing at the Royal Children’s Hospital, Brisbane in April 2014 until pausing recruitment in October 2014 due to the merger of two pediatric hospitals, and relocation to a new hospital. Recruitment recommenced at the Lady Cilento Children’s Hospital, Brisbane in March 2015, completing recruitment in May 2016 when sample size was achieved. The CONSORT flow chart [40] is displayed in Fig. 2, demonstrating enrolment, allocation, follow-up and analysis of the study participants. Ninety-six patients were screened for recruitment, with 68 eligible, and 48 children participating in the pilot study.

### Characteristics

The majority of participants were undergoing treatment for oncological or hematological conditions (n = 39; 81%), with a median age of 5.0 years (IQR 1.8, 11.6) (see Table 1). CVADs were most commonly placed in the internal jugular vein (n = 29; 60%), requiring only a single insertion attempt (n = 44; 94%). The majority received chemotherapy (n = 34; 71%), and around half received antibiotics (n = 23; 48%) during the study period. The majority of participant, CVAD insertion and CVAD utility characteristics were evenly distributed across the intervention groups, with imbalance evident in the frequency of multiple insertion attempts and other characteristics, which is consistent with pilot trial design.

**Table 1:** Participant characteristics (*n* = 48)

	Standard Care	ISD	TA	SSD	Total
Group size	11 (23%)	12 (25%)	12 (25%)	13 (27%)	48 (100%)
Participant characteristics					
Age (years) ^a^	3.1	6.2	4.9	5.0	4.8
	(1.7–14.5)	(1.7–11.7)	(1.0–11.6)	(2.5–8.8)	(1.8–11.6)
Sex (male)	7 (64%)	4 (33%)	8 (67%)	10 (77%)	29 (60%)
Skin integrity:					
good	3 (27%)	4 (33%)	7 (58%)	7 (54%)	21 (44%)
fair	6 (55%)	5 (42%)	4 (33%)	4 (31%)	19 (40%)
poor	2 (18%)	3 (25%)	1 (8%)	2 (15%)	8 (17%)
Skin type (white)	8 (73%)	6 (50%)	10 (83%)	8 (62%)	32 (67%)
Comorbidities:					
none	1 (9%)	0 (0%)	1 (8%)	1 (8%)	3 (6%)
one	7 (64%)	8 (67%)	8 (67%)	9 (69%)	32 (67%)
two or more	3 (27%)	4 (33%)	3 (25%)	3 (23%)	13 (27%)
Diagnosis:					
oncology / hematology	8 (73%)	10 (83%)	10 (83%)	11 (85%)	39 (81%)
medical	2 (18%)	1 (8%)	1 (8%)	1 (8%)	5 (10%)
surgical	1 (9%)	1 (8%)	1 (8%)	1 (8%)	4 (8%)
Infection on recruitment	1 (9%)	2 (17%)	2 (17%)	1 (8%)	6 (12%)
Leucocytes <1,000 / μL	0 (0%)	3 (25%)	1 (8%)	0 (0%)	4 (8%)
CVAD insertion characteristics					
CVAD placement:					
internal jugular	9 (82%)	7 (58%)	7 (58%)	6 (46%)	29 (60%)
subclavian	2 (18%)	4 (33%)	5 (42%)	7 (54%)	18 (38%)
femoral	0 (0%)	1 (8%)	0 (0%)	0 (0%)	1 (2%)
Subsequent insertion	4 (36%)	7 (58%)	5 (42%)	4 (31%)	20 (42%)
No. of lumens:					
one	2 (18%)	0 (0%)	2 (17%)	0 (0%)	4 (8%)
two	8 (73%)	9 (75%)	8 (67%)	11 (85%)	35 (75%)
three	1 (9%)	3 (25%)	2 (17%)	2 (15%)	8 (17%)
Multiple insertion attempts	0 (0%)	0 (0%)	0 (0%)	3 (23%)	3 (6%)
Ultrasound use	4 (36%)	7 (58%)	4 (33%)	4 (31%)	19 (40%)
External length at insertion (cm) ^b^	12.3 (2.5)	15.4 (7.5)	13.3 (3.9)	13.3 (2.0)	13.6 (4.5)
CVAD utility characteristics					
Received continuous intravenous therapy ^c^	4 (36%)	1 (8%)	5 (42%)	6 (46%)	16 (33%)
Received parenteral nutrition and/or lipids ^c^	4 (36%)	4 (33%)	1 (8%)	2 (15%)	11 (23%)
Received chemotherapy ^c^	9 (82%)	8 (67%)	7 (58%)	10 (77%)	34 (71%)
Received blood products ^c^	4 (36%)	4 (33%)	2 (17%)	6 (46%)	16 (33%)
Received antibiotics ^c^	6 (55%)	7 (58%)	1 (8%)	9 (69%)	23 (48%)
Confused, agitated or drowsy ^d^	1 (9%)	3 (25%)	0 (0%)	0 (0%)	4 (8%)
CVAD dwell time (days) ^a, c^	14.1	9.0	8.6	17.1	12.4
	(5.0–27.9)	(4.5–16.5)	(4.1–21.5)	(9.0–27.0)	(5.6–26.0)

n (%) shown unless otherwise noted

*CVAD* Central venous access device*, ISD* Integrated securement dressing*, SSD* Suture-less securement device*, TA* Tissue adhesive*, μL* microlitre

^a^ median (25th and 75th percentiles); percentages may not add up to 100% due to rounding; percentages were calculated using the number of non-missing values in the denominator

^b^ mean and standard deviation

^c^ during study period

^d^ at study completion

### Feasibility of the study process

As displayed in Fig. 2, the majority of feasibility criteria were met with 71% of patients screened eligible, with 22 of these patients missed for recruitment due to CVAD insertion scheduling (weekend, out of hours), and 92% of patients approached for consent agreed to enroll. There were no participants lost to follow up and two patients (4%) withdrew prematurely from the study. Both of these patients withdrew due to skin irritation (itchiness, redness) associated with study products (standard care; ISD). With parental consent, withdrawn participants were included in the analysis due to their prolonged participation leading to the point of withdrawal. Nine participants (19%) deviated from the study protocol for their allocated treatment, however for only 4% of the total studied catheter days (25/647). No primary or secondary outcome data were missed during the study period, and four daily checks were missed out of total 673 (0.6%).

### CVAD failure and complications

**Fig. 3:**
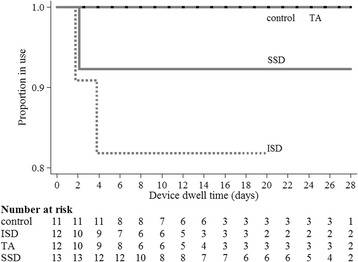
Kaplan-Meier curve of device failure

Six percent of participants (*n* = 3) experienced CVAD failure prior to completion of treatment (IR 4.46 per 1,000 catheter days; 95% CI 1.44–13.8), and 6% of participants (*n* = 3) had a CVAD-associated complication during the study period (IR 4.46 per 1,000 catheter days; 95% CI 1.44–13.8). As described in Table 2, CVAD failure was highest in the ISD group (*n* = 2; 17%), and CVAD complications were highest in the SSD group (*n *= 2; 15%). The TA and standard care groups performed best across both of these criteria with no associated CVAD failures or complications. There were no cases of CVAD-associated thrombosis, complete dislodgement, breakage, local infection, or CVAD-related BSI in any group. CVAD failure results were consistent when failure over time was compared with the Kaplan Meier curve of device failure (Fig. 3).

**Table 2:** Study outcomes (*n* = 48)

	Standard Care	ISD	TA	SSD	Total
Group size	11 (23%)	12 (25%)	12 (25%)	13 (27%)	48 (100%)
CVAD failure					
Failure	0 (0%)	2 (17%)^a^	0 (0%)	1 (8%)^b^	3 (6%)
Catheter-days	162	139	148	224	673
Incidence Rate	0.00 (^)	14.4	0.00 (^)	4.46	4.46
(95% CI)		(3.61–57.7)		(0.63–31.7)	(1.44–13.8)
Log-rank test p-values	referent	0.147	^	0.358	0.280
CVAD complications					
All-cause complications^c^	0 (0%)	1 (8%)	0 (0%)	2 (15%)	3 (6%)
Incidence Rate	0.00 (^)	7.21	0.00 (^)	8.92	4.46
(95% CI)		(1.02–51.2)		(2.23–35.7)	(1.44–13.8)
Log-rank test p-values	referent	0.294	^	0.358	0.600
Complication^c, d^:					
CABSI	0 (0%)	0 (0%)	0 (0%)	1 (8%)	1 (2%)
dislodgement (partial)	0 (0%)	1 (9%)	0 (0%)	1 (8%)	2 (4%)
occlusion (complete)	0 (0%)	0 (0%)	0 (0%)	1 (8%)	1 (2%)
All-cause skin complication ^d, e^	2 (18%)	2 (17%)	0 (0%)	1 (8%)	5 (10%)
Skin complication ^d, e^:					
rash	1 (9%)	0 (0%)	0 (0%)	1 (8%)	2 (4%)
blister	1 (9%)	1 (8%)	0 (0%)	0 (0%)	2 (4%)
itchiness	2 (18%)	1 (8%)	0 (0%)	0 (0%)	3 (6%)
Staff satisfaction ^f, g^					
on application	8.8 (1.6)	7.9 (2.0)	7.9 (2.1)	7.4 (1.7)	8.0 (1.9)
on removal	8.9 (1.6)	8.6 (1.6)	5.5 (2.8)	8.2 (1.6)	7.8 (2.4)
Parent satisfaction on removal ^f, g^	5.3 (2.3)	9.2 (1.0)	8.0 (1.7)	8.5 (1.9)	7.8 (2.3)

n (%) shown unless otherwise noted

*CABSI* Catheter associated bloodstream infection, *CVAD* Central venous access device, *CI* confidence interval, *ISD* Integrated securement dressing, *TA* Tissue adhesive, *SSD* Suture-less securement device

^ = cannot be calculated

^a^ due to spontaneous internal displacement; partial dislodgement

^b^ due to partial dislodgement and occlusion

^c^ at study completion

^d^ Participants could have more than one complication or skin complication

^e^ during the study period

^f^ measured on a 0 (minimum) to 10 (maximum) scale

^g^ mean and standard deviation

### Staff and parental feedback

Overall satisfaction was highest in the integrated securement-dressing (mean 8.5/10; SD 1.2). As described in Table 2, mean staff satisfaction with product application (8.8/10; SD 1.6); and removal (8.9; SD 1.6) was highest for standard care; however mean parent satisfaction at study completion was highest for the ISD arm (9.2/10; SD 1.0).

### Dressing and securement performance

**Fig. 4:**
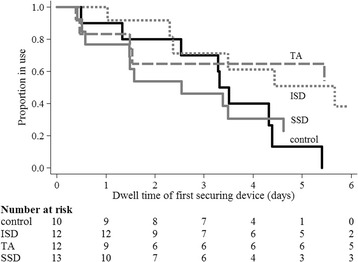
Kaplan-Meier curve of initial securement device failure

Table 3 reports the dressing integrity performance between the study groups. Median time to first dressing change was longest for ISD (95.1 h, IQR 56.1–146), as displayed in the Kaplan Meier curve (Fig. 4), reflecting prolonged time to first dressing change for many participants compared to standard care. Non-routine dressing changes were most common in the standard care arm, with 17 non-routine dressing changes in 162 catheter days (99 per 1000 catheter days). Non-routine dressing changes in the standard care arm were most frequently (59%) due to lifting of the dressing edges.

**Table 3:** Securement device outcomes (*n*=48)

	Standard Care	ISD	TA	SSD
Group size	11 (23%)	12 (25%)	12 (25%)	13 (27%)
Product purchase costs ^a^	$5.12–$6.02	$9.27	$17.57–$18.47	$8.60–$12.32
Patients with dressing changes	10 (91%)	8 (67%)	7 (58%)	13 (100%)
Time to first change (hours) ^b, c^	79.6 (60.8–104)	95.1 (56.1–146)	37.0 (11.2–177)	61.1 (35.4–111)
Reasons for first change:^d^				
routine	1 (10%)	2 (25%)	3 (42%)	3 (8%)
lifting	7 (70%)	2 (25%)	2 (29%)	2 (15%)
sweating	1 (10%)	0	0	0
leakage	2 (20%)	0	0	1 (8%)
skin reaction	1 (10%)	0	0	0
bleeding	6 (60%)	4 (50%)	3 (43%)	8 (61%)
other	1 (10%)	2 (25%)	1 (14%)	0
Non-routine changes	17	10	4	25
Reasons for non-routine changes: ^d^				
lifting	10 (59%)	2 (20%)	2 (50%)	8 (32%)
sweating	1 (6%)	0	0	0
leakage	3 (18%)	0	0	1(4%)
skin reaction	1 (6%)	0	0	0
bleeding	8 (47%)	6 (60%)	3 (74%)	15 (60%)
other	6 (35%)	4 (40%)	1 (25%)	4 (16%)

n (%) shown unless otherwise noted

*ISD* Integrated securement dressing, *TA* tissue adhesive, *SSD* Suture-less securement device, *BPU* bordered polyurethane dressing

^a^ in Australian dollars according to local hospital prices 2016

^b^ median, 25th and 75th percentiles shown

^c^ excluding when initial securing device did not get replaced

^d^ Participants could have more than one reason

### Costs

Each of the experimental arms was more expensive than standard care, when only product purchase costs were considered per application (see Table 3).

## Discussion

This is the world’s first randomized controlled trial evaluating the effectiveness of tunneled CVAD dressing and securement in pediatrics. The trial included innovative securement strategies that had never been tested on this device type, or within this population. This pilot trial evaluated the feasibility of a large efficacy trial, using pre-determined feasibility criteria, a registered and published trial protocol, and rigorous methods.

As per our *a priori* definition of feasibility, a large efficacy trial of tunneled, cuffed CVAD securement and dressing in pediatrics, using these intervention arms, is feasible. Study processes were successful, with targets for eligibility, recruitment, retention, attrition, protocol adherence and missing data achieved. The proportion of failure (6%) and complication (6%) have provided point estimates for future interventional studies; and for a 5% absolute reduction in CVAD failure or complication is to be reached in future efficacy studies (*p* = 0.05; 80% power [39]), 211 participants would be needed for each intervention group. The slow recruitment rate in the pilot trial (48 participants over 21 months), means that conducting the efficacy RCT in a single site would be difficult, and a multisite trial, involving other tertiary pediatric hospitals, would be necessary.

However, of the three CVAD failures within the trial, one was not directly associated with the securement device. Within the ISD group, a CVAD spontaneously internally displaced into a non-central position. This suggests the definition of failure would need amending, to exclude this form of failure, in future trials. Overall, it is too soon to assess if one CVAD dressing and securement option is superior. At this early stage of trialing, all products appear generally safe and feasible for future testing. Our feasibility outcomes demonstrated that high-quality research examining CVAD securement in this population is achievable; however the best product for future research is yet to be established.

The most common cause of CVAD-associated complication within the pilot study was partial dislodgement (2/48; 4%), occurring in two of the intervention arms (ISD, SSD). The rates of complication and failure evident in our study were lower than the recent meta-analysis of observational studies, which found an average CVAD failure proportion of 25% [6], but still demonstrate the problems associated with CVAD management in pediatrics. Despite higher complications rates, parent satisfaction was highest for the ISD arm, with TA and SSD also performing better than standard care.

Within this study both TA and ISD showed potential to increase the time between CVAD insertion and first dressing change, and their true effectiveness and cost-effectiveness should be further investigated. Minimization of dressing changes during the immediate post-insertion period is an important concern for this population. Pediatric patients are frequently non-compliant, and extremely stressed by CVAD dressing changes. Each dressing disruption also potentially places the patient at increased risk for CVAD-associated infection [41] and accidental dislodgement.

Each of the experimental arms was more expensive than standard care, when only basic product purchase costs were considered per application. Additional costs related to the time of skilled clinicians applying and removing the products were not considered, nor the cost of treating complications and failure. These short-term, immediate costs should be weighed against the potential sequelae, if reductions in dressing change frequency, CVAD-associated complications and catheter failure are prevented. Recent case-control studies demonstrated that pediatric CVAD-associated BSIs cost healthcare systems around $55,646 (2011 USD) and 19 additional days admitted to hospital [42], with even higher costs ($69,332; 2011 USD; 21.2 additional days in hospital) for the hematology and oncology pediatric population [43]. Delays to treatment and the insertion of replacement CVADs due to other causes of CVAD failure and complication are also expensive to healthcare systems, and have a significant impact on the morbidity and mortality of pediatric patients. Investment in more expensive CVAD securement devices is likely to be cost-effective if significant improvements in CVAD complication and failure rates can be attained, and future research needs to investigate this.

The recent Cochrane review [9] found moderate quality evidence that chlorhexidine gluconate-impregnated dressing products significantly reduced catheter-related BSI per 1, 000 patient days, and catheter tip colonization, compared with conventional polyurethane dressings. However the majority of these studies were within the adult, critical care population and transferability of these results outside of this population has not been established. Researchers should build on previous work [12, 14] to further examine the role of chlorhexidine gluconate-impregnated dressing products in the oncology and pediatric domain.

CVAD-associated skin injuries were a substantial issue within our study, with site complications identified in 9% of participants, with two additional participants (4%) withdrawing from the study due to skin irritation. This suggests CVAD-associated skin injuries should be included as a primary, rather than a secondary endpoint in future studies. Pediatric CVAD sites are at risk for significant damage due to the combination of skin impairment related to the patient’s age and morbidity, and additional irritation of the CVAD site during maintenance procedures [44]. Local site infection, moisture-related injuries, contact dermatitis and medical adhesive-related injuries (MARSI) related to CVAD sites are frequently reported in the literature [9, 44–48]; however overall prevalence rates have not been published. Identifying patients at risk for skin complication and instituting preventative strategies will further improve clinical outcomes for this vulnerable population.

This pilot study has several limitations. The sample size is small, and the results should not be used to guide clinical practice, however it was never designed to do so. The study was only carried out in two sites in Queensland and its generalizability outside of this population is unknown. Participants, family members and the research staff were not blinded to the intervention allocation, due to the visibility of the securement products. It is highly unlikely that they would intentionally cause CVAD complications because of any preference for any of the treatment arms. The study outcomes of CABSI, catheter-related BSI, and venous thrombosis were assessed by blinded infectious diseases and radiological personnel. The validity and reliability of the study has been ensured through following a prospectively registered study protocol, independent randomization, dedicated ReN, and allocation concealment until study entry.

## Conclusions

High-quality research involving children with tunneled, cuffed CVADs is feasible, and CVAD securement can play an important role in the prevention of CVAD-associated complications [9, 49, 50]. Careful consideration should be given by interdisciplinary clinicians when choosing CVAD securement, to ensure it is the most appropriate device for the individual needs of their patient. Further research is necessary to examine the effectiveness of novel securement and dressing products for CVADs in pediatrics.

## Abbreviations

BPU: Bordered polyurethane
BSI: Bloodstream infection
CABSI: Central venous associated bloodstream infection
CI: Confidence interval
CONSORT: Consolidated standards of reporting trials
CVAD: Central venous access device
HREC: Human research ethics committee
IQR: Interquartile range
IR: Incidence rate
ISD: Integrated securement device
ITT: Intention to treat
RCT: Randomized controlled trial
SD: Standard deviation
SSD: Suture-less securement device
TA: Tissue adhesive
USD: United States Dollar
